# Sensitivity Analysis of Single-Drive, 3-axis MEMS Gyroscope Using COMSOL Multiphysics

**DOI:** 10.3390/mi11121030

**Published:** 2020-11-24

**Authors:** Hussamud Din, Faisal Iqbal, Byeungleul Lee

**Affiliations:** School of Mechatronics Engineering, Korea University of Technology and Education, Cheonan 31253, Korea; hussam@koreatech.ac.kr (H.D.); faisal@koreatech.ac.kr (F.I.)

**Keywords:** MEMS, gyroscope, mechanical sensitivity, finite element analysis (FEA) model, COMSOL

## Abstract

In this paper, a COMSOL Multiphysics-based methodology is presented for evaluation of the microelectromechanical systems (MEMS) gyroscope. The established finite element analysis (FEA) model was successfully validated through a comparison with analytical and Matlab/Simulink analysis results. A simplified single-drive, 3-axis MEMS gyroscope was analyzed using a mode split approach, having a drive resonant frequency of 24,918 Hz, with the x-sense, y-sense, and z-sense being 25,625, 25,886, and 25,806 Hz, respectively. Drive-mode analysis was carried out and a maximum drive-displacement of 4.0 μm was computed for a 0.378 μN harmonic drive force. Mechanical sensitivity was computed at 2000 degrees per second (dps) input angular rate while the scale factor for roll, pitch, and yaw was computed to be 0.014, 0.011, and 0.013 nm/dps, respectively.

## 1. Introduction

The microelectromechanical systems (MEMS) vibrating gyroscope is used to measure the rotation rate of a body based on the Coriolis effect. The MEMS gyroscope has various advantages over the conventional gyroscope, such as its light weight, compact size, economical aspect, and easy integration with mainstream complimentary metal-oxide-semiconductor (CMOS) technology. The MEMS gyroscope has a variety of applications in the area of consumer electronics, navigation systems, automobiles and robot control [1,2,3].

Over the past few decades, intensive research has been carried out to improve the performance of MEMS gyroscopes. This research journey started from a single-axis MEMS gyroscope capable of measuring angular rate in only one axis [4,5], and extended to a single-drive, 3-axis MEMS gyroscope which can measure the angular rate in all three axes [2,6,7,8,9]. The working principle of any kind of MEMS vibratory gyroscope is based on the Coriolis effect, to realize the energy transfer between the drive and sense resonant modes [10,11,12]. To meet today’s demanding industry specifications, the sound performance of the gyroscope is vital, particularly in terms of sensitivity and accuracy [13,14]. MEMS products have relatively more manufacturing uncertainties compared with macro-scale products. Such uncertainties affect the performance of the MEMS products [15,16]. Therefore, performance evaluation is necessary before fabrication to achieve a reliable design for the MEMS gyroscope.

Many efforts have been made by researchers using system-level analysis and a finite element analysis (FEA) simulation approach to estimate the mechanical sensitivity of the MEMS gyroscope. A MEMS tuning fork gyroscope (TFG) with an anchored leverage mechanism was designed and analyzed for mechanical sensitivity using the ANSYS Workbench [14]. Three types of mechanical structures and their different spring stiffnesses were analyzed to improve the mechanical sensitivity. The computed mechanical sensitivity was validated theoretically and showed improvement by 79.10%, 81.33%, and 68.06%, respectively. A single-axis, in-plane MEMS vibratory rate gyroscope was simulated for sensitivity estimation using CoventorWare software. A maximum Coriolis displacement of 424 nm was computed by applying an input angular rate of 400 degrees per second (dps), resulting in a 1.06 nm/dps scale factor [17]. A ring gyroscope was analyzed and a maximum driving displacement of 2.91 μm, along with a mechanical sensitivity of 3.6 nm/dps, was computed using the ANSYS Workbench [18]. System level simulations were carried out for the drive-mode and sense-mode operations of a single-drive, 3-axis MEMS gyroscope [19]. A system-level simulation approach was presented to estimate the effect of imperfection and fabrication errors in the MEMS gyroscope [20]. The transient and steady responses for sense-modes, and response to angular rate were performed for a fence structured MEMS gyroscope through system level simulations in PSpice [21]. All the aforementioned researches were either about a single-axis gyroscope or were limited to theoretical and system-level approaches.

This paper focuses on the development of an FEA simulation-based methodology, using the solid mechanics module of COMSOL Multiphysics, which can be used for the evaluation of a single-drive, 3-axis MEMS gyroscope, as well as a single-axis gyroscope. In the gyroscope development process, FEA simulation is one of the powerful and important tools to determine the design parameters and it is necessary for robust optimization [22,23]. Cross-axis sensitivity and quadrature error evaluation for a single-drive, 3-axis MEMS gyroscope is difficult through analytical or Matlab/Simulink analysis. However, COMSOL Multiphysics provides an easier approach for these estimations, which is comparatively more realistic and informative than other approaches. A simplified design for a single-drive, 3-axis MEMS gyroscope presented in a previous work [24] is analyzed in this study. That simplified design of a single-drive, 3-axis MEMS gyroscope was simulated employing the established FEA methodology, and mechanical sensitivity was computed. The simulation results were validated by comparing them with system-level and analytical results, respectively.

This paper is structured as follows. In Section 2, the mechanical structure and the working principle of a single-drive, 3-axis MEMS gyroscope are presented. In Section 3, the proposed methodology is discussed in detail, including modal analysis, drive-analysis, and mechanical sensitivity analysis using three approaches, followed by their comparison. The paper is concluded in Section 4.

## 2. Mechanical Structure and Working Principle

**Figure 1:**
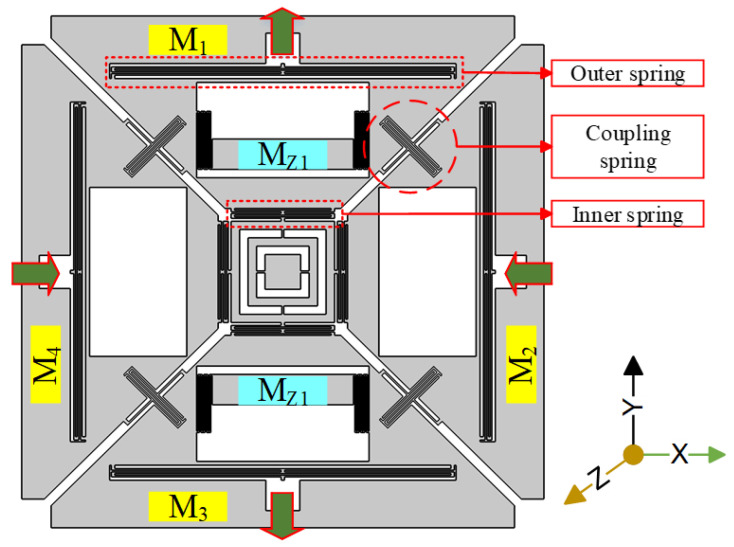
Mechanical structure of the proposed single-drive, 3-axis microelectromechanical systems (MEMS) gyroscope.

The mechanical structure of the proposed single-drive, 3-axis MEMS gyroscope consisted of four drive masses named M_1_, M_2_, M_3_, and M_4_, suspended in the x-y plane, as shown in Figure 1. These masses were coupled by a unique coupling mechanism consisting of one z-shaped coupling spring. This type of coupling spring has the quality to suppress the unwanted modes of drive masses while keeping the target sense modes close to the drive-mode. In the drive-scheme of the proposed design, M_1_ and M_3_, which were opposite to each other, moved outward, while the two other opposite masses, M_2_ and M_4_, moved inward. This kind of drive-scheme was capable of reducing the slide film damping [10]. There were two z-sense masses named M_z1_ and M_z2_ as shown in Figure 1. There were eight lateral double-folded springs, four outside and four inside the structure. The outer double-folded springs were anchored at one point to the substrate and the other sides were connected at two points to each mass, providing an out-of-plane motion from the outside. The roll and pitch modes exhibited out-of-plane motion, whereas the yaw mode exhibited in-plane motion.

The working principle of the vibrating MEMS gyroscope is based on the Coriolis effect. Whenever the driving masses are driven at resonance and an input angular rate is applied to a movable sense-mass, the sense-mass experiences a force called the Coriolis force. This Coriolis force generates a displacement in the movable sense-mass perpendicular to the direction of drive motion, as well as to the axis of rotation, called sense-displacement.

## 3. Methodology

### 3.1. Modal Analysis

**Figure 2:**
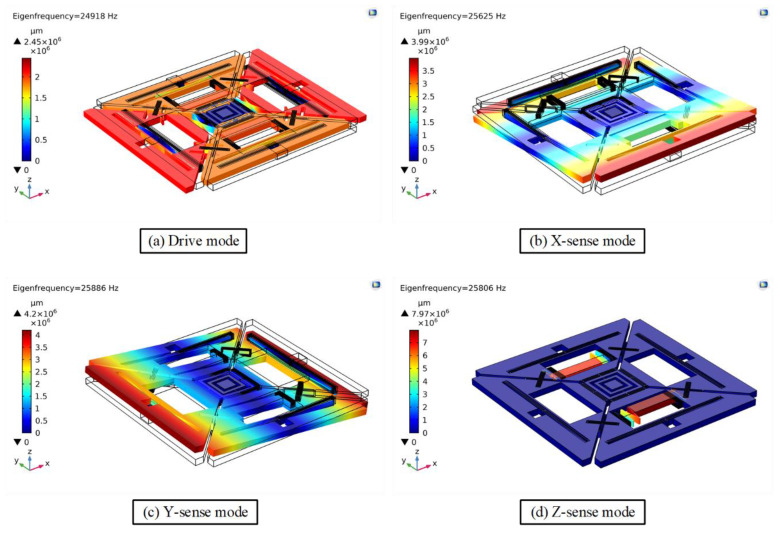
Resonant frequency mode shapes of the proposed single-drive, 3-axis MEMS gyroscope.

The modal analysis of the MEMS vibratory gyroscope determines its eigen frequencies and mode shapes. Eigen frequencies and their mode shapes are important to be determined before further evaluation of the MEMS gyroscope. The simulation results of modal analysis for the proposed design of a single-drive, 3-axis MEMS gyroscope are presented in Table 1 and Figure 2, respectively.

Figure 2 shows that the drive-mode exhibits the same scheme as discussed in Section 2, where two opposite masses (i.e., M_1_ and M_3_) move outward while the two other masses (M_2_ and M_4_) move inward. Similarly, x/y-sense motions are out of the plane while the z-sense exhibits in-plane motion following the same scheme as discussed in Section 2. Hence, when the gyroscope is driven at a driving resonant frequency and the sensitive axes encounter an input angular rate, Ω, the sensitive masses will be excited in the modes as shown in Figure 2.

### 3.2. Drive-Mode Analysis

**Figure 3:**
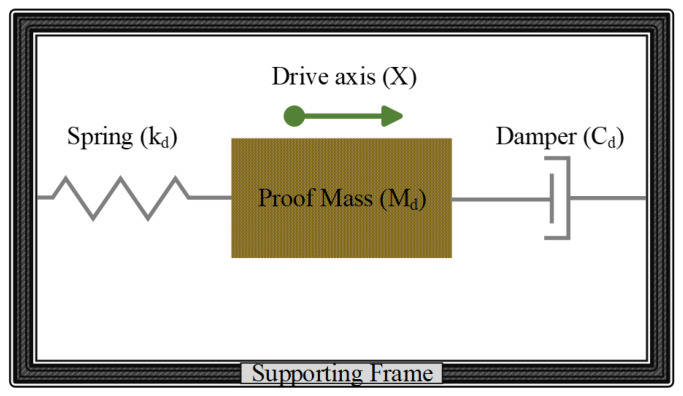
One degree of freedom (1-DOF) mass spring damper system.

A typical MEMS vibratory rate gyroscope consists of a proof mass suspended by elastic beams driven by harmonic oscillations to provide momentum for the Coriolis effect. The drive-mode of a MEMS vibratory gyroscope is a simple one degree of freedom (1-DOF) resonator, which is equivalent to a mass-spring damper system as shown in Figure 3. The drive-mode motion is described by Equation (1), in which the system is excited by a sinusoidal drive force.

(1) $$M_{d}\ddot{x_{d}}+C_{d}\dot{x_{d}}+K_{d}x_{d}=F_{d}\sin\omega t.$$

Here, $M_{d},$ $C_{d}$, $K_{d}$, and $F_{d}$ represent the drive mass, damping coefficient, spring stiffness, and sinusoidal drive-mode excitation force, respectively, while $\ddot{x_{d}}$, $\dot{x_{d}}$, and $x_{d}$ represent the drive-mode acceleration, velocity, and displacement, respectively. The drive-mode resonant frequency is given by $\omega_{d}=\sqrt{K_{d}/M_{d}}$ and the quality factor $Q_{d}=M_{d}\omega_{d}/C_{d}$. Practically all vibratory rate gyroscopes operate on the drive-mode resonant frequency, having a drive-mode displacement phase of −90° at resonance. The maximum drive-mode displacement amplitude $x_{d_{\max}}$ and phase $\phi_{d}$ are given by Equations (2) and (3), respectively, where ω is the drive force frequency, which is equal to the drive resonant frequency $\omega_{d}$ [25].

(2) $$x_{d_{\max}}=\frac{Q_{d}F_{d}}{\omega_{d}^{2}M_{d}}$$

(3) $$\phi_{d}=-\tan^{-1}\frac{\frac{\omega }{Q_{d}\omega_{d}}}{1-{\left(\frac{\omega }{\omega_{d}}\right)}^{2}}≅-\pi /2$$

In this work, the proposed single-drive, 3-axis MEMS gyroscope was driven by a harmonic driving force in COMSOL Multiphysics using the frequency domain study. The simulated frequency response of the gyroscope showed the drive-mode displacement amplitude and phase as a result of applied driving force. To enhance the accuracy of the results, Rayleigh or material damping was added in the model to obtain the frequency response of the system. Rayleigh damping was proportional to the linear combination of mass and stiffness. The damping parameter “C_d_” was related to the mass and stiffness of the system and required two material constants, α_dM_ and β_dK_. The values of these constants can be derived from the damping ratio of the system, which was the percentage of critical damping [26,27]. The drive quality factor $Q_{d}$ used in this study was assumed as 10,000 [10,25], while the remaining parameters used in this study are listed in Table 2.

**Figure 4:**
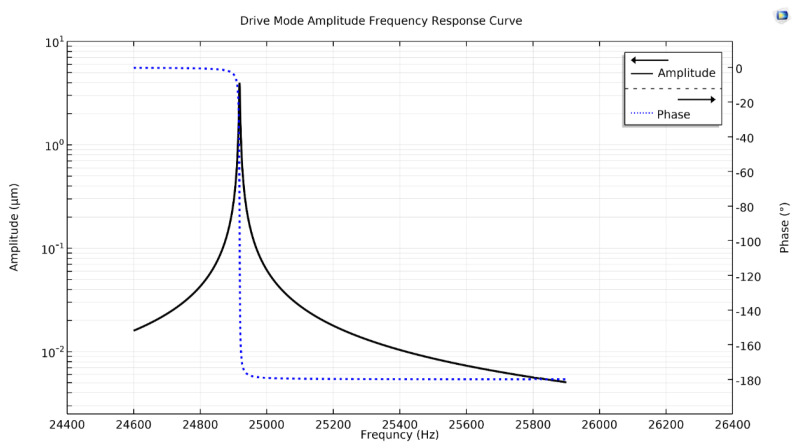
Drive-mode amplitude frequency response curve of the proposed single-drive, 3-axis MEMS gyroscope.

The drive excitation force $F_{d}$ was differentially applied as a boundary load in one axis of the gyroscope as the other axis of the proposed single-drive, 3-axis MEMS gyroscope structure was being coupled. A parametric sweep for a drive frequency of 24,918 Hz in a frequency band of 24,600 to 25,900 Hz, having 1 Hz resolution, was added in the study settings. The simulated frequency response was plotted in Figure 4, depicting a 4.0 μm peak amplitude at a drive resonant frequency of 24,918 Hz as the drive-displacement, with a phase of $\phi_{d}=-90{}^{\circ}$. This validated the feasibility of the drive-mode operation and structure of the single-drive, 3-axis MEMS gyroscope.

### 3.3. Mechanical Sensitivity Analysis

Sensitivity of the MEMS vibratory gyroscope was comprised of two factors; i.e., peripheral circuit gain and mechanical sensitivity. For better understanding, this study only focuses on the mechanical sensitivity of the single-drive, 3-axis MEMS gyroscope evaluated in COMSOL Multiphysics. To validate the established COMSOL model, FEA simulated results were compared with analytical and system level approach results.

For sensitivity estimation, it is necessary to find the Coriolis response of the MEMS gyroscope. Sense-mode of the gyroscope is coupled with drive-mode through Coriolis force $F_{C}$, given by Equation (4).

(4) $$F_{C}=-2M_{C}\Omega \dot{x_{d}}=-2M_{C}{\Omega \omega }_{d}x_{d_{\max}}\cos\left(\omega_{d}t+\phi_{d}\right)$$

In this equation, $M_{C}$ is Coriolis mass, which is equal to $M_{d}$ in a single mass system. To amplify the mechanical response of the gyroscope at its resonant frequency, its sense-mode oscillator is also modeled as a 1-DOF resonator which is governed by Equation (5).

(5) $$M_{s}\ddot{y_{s}}+C_{s}\dot{y_{s}}+K_{s}y_{s}=-2M_{C}{\Omega \omega }_{d}x_{d_{\max}}\cos\left(\omega_{d}t+\phi_{d}\right)$$

Here, $M_{s}$, $C_{s}$, $K_{s}$, $\ddot{y_{s}}$, $\dot{y_{s}}$ and $y_{s}$ are the sense-mode mass, damping coefficient, spring stiffness, acceleration, velocity, and displacement, respectively. It is notable that in a single mass system, the terms $M_{C}$, $M_{d}$, and $M_{s}$ are equal. The sense-mode resonant frequency and the quality factor are given by $\omega_{s}=\sqrt{K_{s}/M_{s}}$ and $Q_{s}=M_{s}\omega_{s}/C_{s}$, respectively, while the sense-mode amplitude is given by Equation (6).

(6) $$y_{s_{\max}}=\frac{2{\Omega \omega }_{d}x_{d_{\max}}}{\sqrt{{\left[\omega_{s}^{2}-\omega_{d}^{2}\right]}^{2}+{\left[\frac{\omega_{d}}{Q_{s}\omega_{s}}\right]}^{2}}}$$

When the drive-mode oscillator is operated at a drive resonant frequency, which gives a drive-displacement peak with a phase −90° relative to input drive force signal. The sense-displacement phase of the gyroscope is independent of $\phi_{d}$ and dependent on $\omega_{d}$, $Q_{s}$ and the frequency separation, “Δf”, between the drive and sense modes and can be described by Equation (7).

(7) $$\phi_{s}=\phi_{d}-90{}^{\circ}-\tan^{-1}\frac{1/Q_{s}(\omega_{d}/(\omega_{d}+\Delta f))}{1-{\left(\omega_{d}/(\omega_{d}+\Delta f)\right)}^{2}}$$

As we know that, the drive displacement is −90° phase-shift from the driving force, and the Coriolis sense-displacement phase depends on the Coriolis force phase, which is same as the drive velocity phase [25]. Sense-displacements of the proposed single-drive, 3-axis MEMS gyroscope are calculated using Equation (6), utilizing the resonant frequencies computed by modal analysis in COMSOL and the parameters listed in Table 3. The sense quality factor $Q_{s}$ used in this study was assumed as 1000 [10,25]. The calculated sense-displacements of all the three axes are listed in Table 4.

### 3.4. FEA Simulation Approach

Mechanical sensitivity of the single-drive, 3-axis MEMS gyroscope was computed in COMSOL Multiphysics using a frequency-domain study. To measure the Coriolis response or sense displacement of the proposed design, it was necessary to apply the input angular rate to the structure after it was driven at a drive-resonant frequency. In a frequency-domain study, rotatory frame was added to the COMSOL Multiphysics model in order to apply an angular rate and to generate a Coriolis force. A drive frequency sweep of 24,600 to 25,900 Hz at 1 Hz resolution was added to the study. An auxiliary sweep for the input angular rate of 2000 dps was also added in the study settings and applied through a rotatory frame in each axis while a frequency response function was computed.

**Figure 5:**
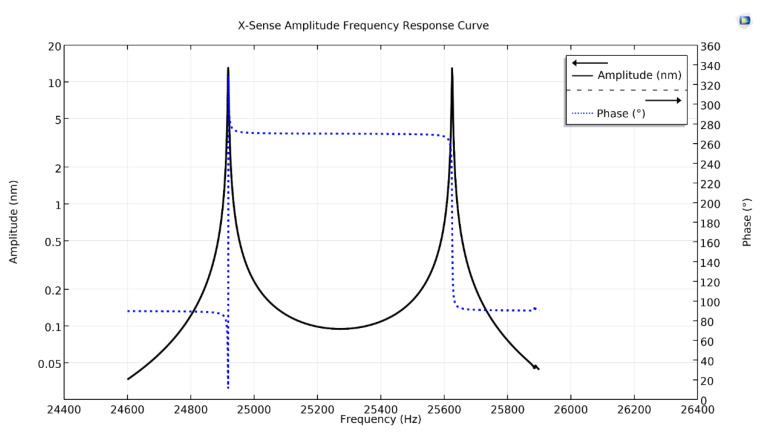
X-sense amplitude frequency response curve of the proposed single-drive, 3-axis MEMS gyroscope.

Figure 5 shows the x-axis Coriolis response plot of the proposed design, as the input angular rate was applied in the x-axis through the rotatory frame. There are two amplitude peaks in the plot, the first peak having an amplitude of 13.43 nm with a phase-shift of −90°, while the second peak has an amplitude of 13.0 nm with a phase-shift of 180°. The first peak at drive resonant frequency is due to the mode-split condition, while the second peak at the Coriolis frequency is due to the mode-match condition.

**Figure 6:**
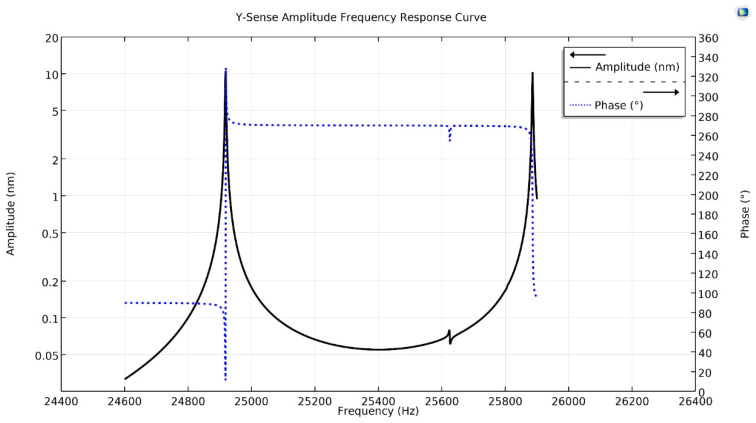
Y-sense amplitude frequency response curve of the proposed single-drive, 3-axis MEMS gyroscope.

**Figure 7:**
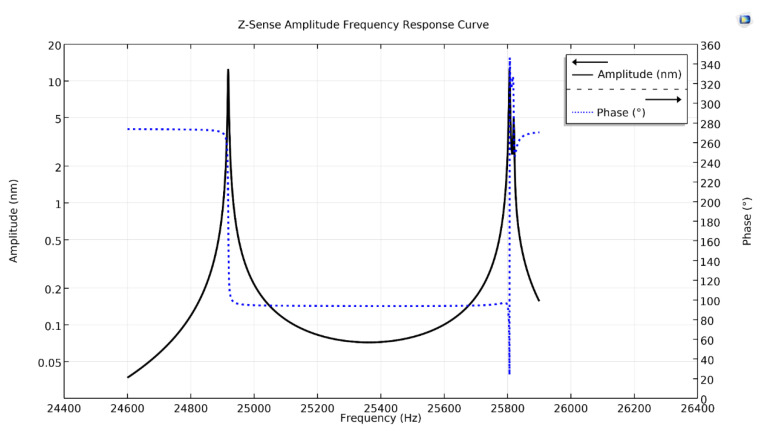
Z-sense amplitude frequency response curve of the proposed single-drive, 3-axis MEMS gyroscope.

Figure 6 shows the y-Coriolis response plot, having two peaks along with their phases. The first peak at a drive resonant frequency, having peak amplitude of 10.60 nm with a phase-shift of −90°, while the second on the Coriolis frequency, having peak amplitude of 10.2 nm with a phase-shift of 180°. Similarly, Figure 7, shows z-Coriolis response plot having two peaks. The first peak is on the drive resonant frequency with an amplitude of 12.28 nm having phase-shift of −90°, while the second peak is on the Coriolis frequency and has an amplitude of 12.4 nm, with a phase-shift of 180°. As the sense-mode frequencies are higher than the drive-mode frequency, all the sense-displacements of the proposed design are at a −90° phase-shift from the drive-mode [25].

Analytical and COMSOL data for sense-displacements of the proposed single-drive, 3-axis MEMS gyroscope were compared and the percentage error was calculated in order to validate the COMSOL model, as summarized in Table 5. The comparison of both data provides an error estimation which leads to an interesting factor in MEMS, called quadrature error. The quadrature error estimation for a single-drive, 3-axis MEMS gyroscope is difficult to determine using an analytical or a Matlab/Simulink model, due to its structure complexity; however, COMSOL provides an easier approach. Quadrature errors for the proposed single-drive, 3-axis MEMS gyroscope were computed using the established FEA methodology at zero-rate input in their respective axes. For the proposed single drive 3-axis MEMS gyroscope, the computed quadrature error is higher in the z-axis compared with the x/y-axes. The comparative error between COMSOL and analytical results, after compensating for the quadrature error, was reduced and the data is summarized in Table 6.

**Figure 8:**
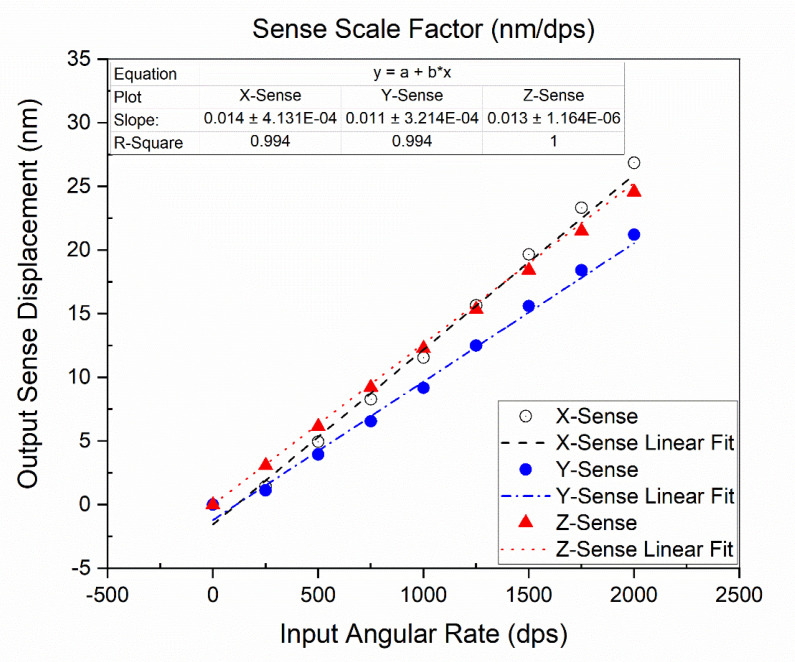
Scale factor plot of the proposed single-drive, 3-axis MEMS gyroscope.

To measure the scale factor of the proposed design, the frequency domain analysis was carried out at a fixed drive resonant frequency of 24,918 Hz, for mode-split condition operation, and an auxiliary sweep for input angular rate of 0 to 2000 dps was applied. Sense-displacement for each axis was computed differentially by applying the input angular rate in its respective axis through the rotatory frame. The computed differential sense-displacements for x/y-axis and z-axis, are listed in Table 7. The FEA simulation results scale factor for the x-sense, y-sense, and the z-sense are 0.014, 0.011, and 0.013 nm/dps, respectively, as shown in Figure 8.

### 3.5. System Level Analysis

**Figure 9:**
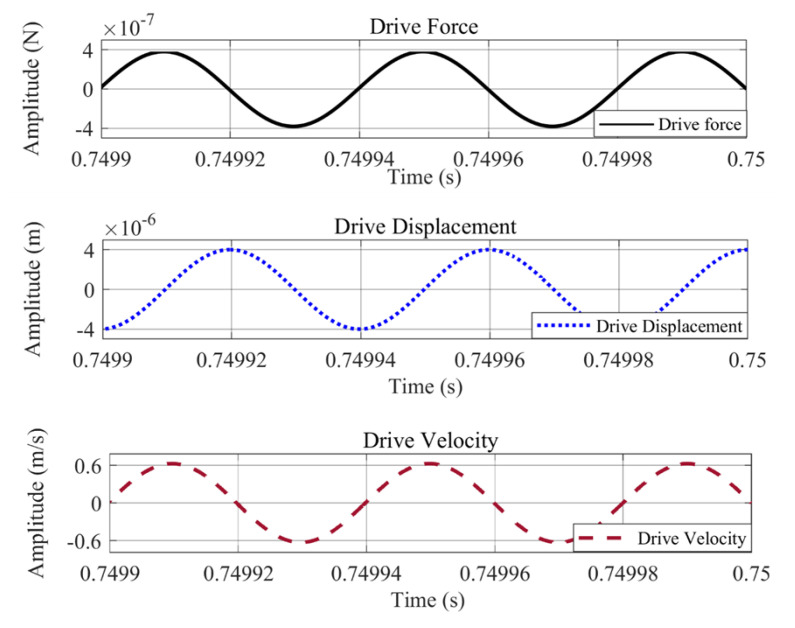
Drive-mode analysis of the proposed single-drive, 3-axis MEMS gyroscope using Matlab/Simulink.

**Figure 10:**
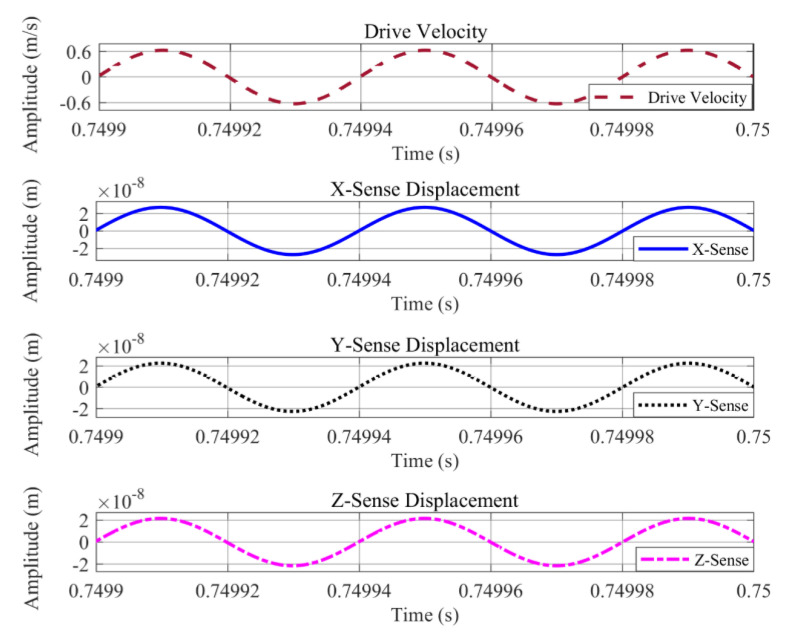
Sense-mode analysis of the proposed single-drive, 3-axis MEMS gyroscope using Matlab/Simulink.

Simulations were carried out in Matlab/Simulink for the drive and sense-mode operations of the proposed single-drive, 3-axis MEMS gyroscope. The Simulink model for this analysis consisted of an open loop drive and sense detection schemes. The same harmonic driving force was applied to the system at a drive-resonant frequency and the drive force signal was achieved along with the drive-displacement amplitude and velocity. The drive analysis is shown in Figure 9, which illustrates that the drive-displacement has 4.0 μm amplitude with a −90° phase-shift from the driving force signal. For the Coriolis response or sense-displacement analysis, an input angular rate of 2000 dps or 34.91 rad/s was applied and the sense signal was achieved showing sense-displacement plots which exhibit −90° phase-shift from drive-displacement. The parameters used for this analysis are listed in Table 8. Sense-displacements for the x-axis, y-axis, and z-axis are shown in Figure 10, having amplitudes of 27.087, 22.52, and 21.49 nm, respectively. These results were compared with analytically calculated results and COMSOL simulation results and are listed in Table 9.

The Simulink analysis results and analytical model results validated the COMSOL Multiphysics model for measuring the mechanical sensitivity of the single-drive, 3-axis MEMS gyroscope.

## 4. Conclusion and Future Work

In this paper, the FEA simulation methodology using COMSOL Multiphysics was established to evaluate the mechanical sensitivity of single-drive, 3-axis MEMS gyroscope. Driving analysis and sensitivity analysis for the single-drive, 3-axis MEMS gyroscope were carried out using the established FEA model in COMSOL Multiphysics. The proposed method was successfully validated by comparison with analytical and Matlab/Simulink model results. The proposed FEA model provided an easy and informative approach for evaluation of the 3-axis, as well as the single-axis MEMS gyroscope, compared with analytical and Matlab/Simulink approaches. Scale factors of the proposed single-drive, 3-axis MEMS gyroscope for the x-axis, y-axis, and z-axis were computed as 0.014, 0.011, and 0.013 nm/dps, respectively, using the FEA model. The proposed FEA model provided information about the quadrature errors which were difficult to design analytically or in a system-level approach for the single-drive, 3-axis MEMS gyroscope due to its complex mechanical structure compared with a single-axis MEMS gyroscope. The reported results and validation of the proposed FEA model prove that this method is more realistic compared with other approaches. Moreover, the proposed FEA model is feasible to estimate the cross-axis sensitivity in a single-drive, 3-axis MEMS gyroscope, which is not possible at a system-level design. Likewise, this model is helpful for researchers in the designing and optimization of single-drive, 3 axis MEMS gyroscope as well as in its performance evaluation. In MEMS research, the fabrication process and experimental work are the expensive and time consuming phases. However, to minimize the cost and time of the research process, the proposed FEA simulation methodology can be employed for a wide range of MEMS gyroscopes based on Coriolis-force, for evaluation and design optimization. Cross-axis sensitivity and fabrication errors were not considered in this work, which will be evaluated in our future work, along with further analysis on quadrature error.

## Figures and Tables

**Table 1:** Resonant frequencies of the proposed single-drive, 3-axis MEMS gyroscope.

Frequency Mode		Frequency (Hz)	Frequency Difference (Hz)
f_dr_	Drive mode	24918	-
f_cx_	x-sense	25625	$f_{\mathrm{dr}}-f_{\mathrm{cx}}=-707$
f_cz_	z-sense	25806	$f_{\mathrm{dr}}-f_{\mathrm{cz}}=-888$
f_cy_	y-sense	25886	$f_{\mathrm{dr}}-f_{\mathrm{cy}}=-968$

**Table 2:** Drive-mode analysis parameters for the proposed single-drive, 3-axis MEMS gyroscope.

Parameters		Value	Remarks
$Q_{d}$	Drive quality factor	10,000	Assumption
$M_{d}$	Drive mass	$3.86\times 10^{-8}\;\mathrm{kg}$	Simulation
$f_{\mathrm{dr}}$	Drive frequency	24,918 Hz	Simulation
$F_{d}$	Drive force	0.378 μN	Analytical
$\alpha_{\mathrm{dM}}$	$4{\pi f}_{d}/3Q_{d}$	10.425 Hz	Damping parameters from COMSOL
$\beta_{\mathrm{dK}}$	$1/6{\pi f}_{d}Q_{d}$	$2.13\times 10^{-10}\;s$	

**Table 3:** Sense-mode analysis parameters for the proposed single-drive, 3-axis MEMS gyroscope.

Parameters		Value	Remarks
$Q_{s}$	Sense quality factor	1000	Assumption
$x_{d_{\max}}$	Drive displacement	4.0 μm	Drive displacement in x-axis
		3.5 μm	Drive displacement in y-axis
Ω	Angular rate	2000 dps	Input angular rate

**Table 4:** Analytically calculated sense displacements of the proposed single-drive, 3-axis MEMS gyroscope.

Parameters		Value (nm)	Remarks
$y_{s_{\mathrm{maxA}}}$	$y_{s_{\mathrm{xA}}}$	27.087	x-sense displacement
	$y_{s_{\mathrm{yA}}}$	22.52	y-sense displacement
	$y_{s_{\mathrm{zA}}}$	21.49	z-sense displacement

**Table 5:** Analytical and FEA simulated sense-displacements comparison.

Parameters	Analytical Value	COMSOL Value	Percentage Error
$y_{s_{x}}\;\left(\mathrm{nm}\right)$	27.087	26.87	0.80%
$y_{s_{y}}\;\left(\mathrm{nm}\right)$	22.52	21.21	5.82%
$y_{s_{z}}\;\left(\mathrm{nm}\right)$	21.49	24.57	−14.33%

**Table 6:** Quadrature error estimation in sense-displacement.

Parameters	Analytical Value	Quadrature Error	COMSOL Value without Q. Error	Percentage Error without Q. Error
$y_{s_{x}}\;\left(\mathrm{nm}\right)$	27.087	0.0015	26.87	0.81%
$y_{s_{y}}\;\left(\mathrm{nm}\right)$	22.52	0.0013	21.21	5.82%
$y_{s_{z}}\;\left(\mathrm{nm}\right)$	21.49	2.781	21.79	−1.40%

**Table 7:** Computed sense-displacements of the proposed single-drive, 3-axis MEMS gyroscope.

Parameters		Value (nm)	Remarks
$y_{s_{\mathrm{maxC}}}$	$y_{s_{\mathrm{xC}}}$	26.87	x-sense displacement
	$y_{s_{\mathrm{yC}}}$	21.21	y-sense displacement
	$y_{s_{\mathrm{zC}}}$	24.57	z-sense displacement

**Table 8:** Parameters for Matlab/Simulink analysis of the proposed single-drive, 3-axis MEMS gyroscope.

Parameter	Value	Remarks
$M_{d}$	$3.86\times 10^{-8}\;\mathrm{kg}$	Drive mass
$F_{d}$	0.378 μN	Drive force
$M_{\mathrm{xs}}$	$2.045\times 10^{-8}\;\mathrm{kg}$	x-sense mass
$M_{\mathrm{ys}}$	$1.815\times 10^{-8}\;\mathrm{kg}$	y-sense mass
$M_{\mathrm{zs}}$	$1.15\times 10^{-9}\;\mathrm{kg}$	z-sense mass
$Q_{d}$	10000	Drive quality factor
$Q_{s}$	1000	Sense quality factor
Ω	2000 dps	Input-angular rate

**Table 9:** Sense-displacement comparison of the proposed single-drive, 3-axis MEMS gyroscope.

Parameter	Analytical	COMSOL	Simulink	Remarks
$y_{s_{x}}\;\left(\mathrm{nm}\right)$	27.087	26.87	27.087	x-sense displacement
$y_{s_{y}}\;\left(\mathrm{nm}\right)$	22.52	21.21	22.52	y-sense displacement
$y_{s_{z}}\;\left(\mathrm{nm}\right)$	21.49	24.57	21.49	z-sense displacement
